# The role of the MAP kinase−kinase protein StMKK1 in potato immunity to different pathogens

**DOI:** 10.1038/s41438-021-00556-5

**Published:** 2021-06-01

**Authors:** Xiaokang Chen, Wenbin Wang, Pingping Cai, Ziwei Wang, Tingting Li, Yu Du

**Affiliations:** College of Horticulture, Northwest A&F University and Shaanxi Engineering Research Center for Vegetables, Yangling, Shaanxi 712100 China

**Keywords:** Plant immunity, Plant stress responses

## Abstract

Mitogen-activated protein kinase (MAPK) cascades play important roles in plant immunity. Previously, we reported that the potato StMKK1 protein negatively regulates *Nicotiana benthamiana* resistance to *Phytophthora infestans*. However, the functions of StMKK1 in potato immunity are unknown. To investigate the roles of StMKK1 in potato resistance to different pathogens, such as the potato late-blight pathogen *P. infestans*, the bacterial wilt pathogen *Ralstonia solanacearum*, and the gray-mold fungal pathogen *Botrytis cinerea*, we generated *StMKK1* transgenic lines and investigated the response of potato transformants to destructive oomycete, bacterial, and fungal pathogens. The results showed that overexpression and silencing of *StMKK1* do not alter plant growth and development. Interestingly, we found that StMKK1 negatively regulated potato resistance to the hemibiotrophic/biotrophic pathogens *P. infestans* and *R. solanacearum*, while it positively regulated potato resistance to the necrotrophic pathogen *B. cinerea*. Further investigation showed that overexpression of *StMKK1* suppressed potato pathogen-associated molecular pattern (PAMP)-triggered immunity (PTI) and salicylic acid (SA)-related responses, while silencing of *StMKK1* enhanced PTI and SA-related immune responses. Taken together, our results showed that StMKK1 plays dual roles in potato defense against different plant pathogens via negative regulation of PTI and SA-related signaling pathways.

## Introduction

Potato, the world’s third most important food crop (FAOSTAT 2013), is threatened by a group of major pathogens, *Phytophthora infestans*, *Ralstonia solanacearum*, and *Botrytis cinerea*. *P. infestans*, a hemibiotrophic pathogenic oomycete^1^, is the causal agent of potato late blight, which results in global costs of more than 6 billion dollars per year^2^. The biotrophic bacterial pathogen *R. solanacearum* causes one of the most notorious diseases of potato, known as potato bacterial wilt^3^. Potato gray mold is a common disease and is caused by the necrotrophic fungal pathogen *B. cinerea*^4^. These diseases lead to billions of dollars of potato production losses annually and pose serious threats to food security.

To combat pathogens, plants have evolved two-layered plant immune systems^5^. The first layer relies on pattern-recognition receptors (PRRs), which can percept pathogen-associated molecular patterns (PAMPs). The perception of PAMPs triggers a series of defense responses called PAMP-triggered immunity (PTI)^6^. The second layer is mediated by plant-resistance (R) proteins that can detect cognate pathogen effectors and subsequently activate a robust immune response called effector-triggered immunity (ETI)^7^.

The mitogen-activated protein kinase (MAPK) cascade is an important pathway that transduces extracellular stimuli into intracellular responses^8,9^. MAPK cascades generally contain three kinase components, MAP kinase kinase kinase (MAPKKKs), MAP kinase kinase (MAPKKs), and MAPKs^10^. It has been shown that the activation of both PRR proteins and R proteins can induce MAPK cascades, which play central roles in signaling defense responses^11–13^. For example, MEKK1−MKK4/MKK5−MPK3/MPK6, which can be activated by the bacterial PAMP flg22, could induce *WRKY* transcription factor gene expression and positively regulate plant defense against bacterial and fungal pathogens^14,15^. In addition, another well-demonstrated MAPK cascade in *Arabidopsis*, consisting of MKK1/MKK2 and MPK4, negatively regulates plant immunity by regulating the expression of *PR* genes and the accumulation of H_2_O_2_^16^. In addition, AtMKK3, which can enhance the expression of *PR* genes, was shown to play a role in the defense against *Pseudomonas syringae* pv. *tomato DC3000*^17^.

MKKs play dual roles in plant defense against different pathogens. For example, overexpression of *GhMKK1* decreases *N. benthamiana* resistance to *R. solanacearum*^18^, while ZmMKK1 positively regulates *N. tabacum* resistance to *Pseudomonas solanacearum*^19^. Although the role of several MKK proteins in plant immunity has already been studied in *Arabidopsis*, their role in the potato response to different classes of pathogens is still unknown. Several MKK proteins were reported to play a role in SA-related immune signaling^20^. For example, tomato SlMKK2 and SlMKK4 were shown to be involved in both JA- and SA-signaling pathways^21^. Constitutively active AtMKK2-EE was shown to reduce SA levels upon *P. syringae* infection^22^.

Previously, we showed that the potato StMKK1 protein is a host target of the *P. infestans* RXLR effector. Overexpression of *StMKK1* in *N. benthamiana* promotes plant susceptibility to *P. infestans*, indicating that StMKK1 negatively regulates plant immunity to the late-blight pathogen. However, the functions of StMKK1 in potato to *P. infestans* and other pathogens remain unknown. In this study, to dissect the role of StMKK1 in potato resistance to different plant pathogens, we constructed *StMKK1* transgenic potato and investigated the response of potato transformants to oomycete, fungal, and bacterial pathogens.

## Results and discussion

### Phylogenetic analysis of StMKK1

*Arabidopsis* encodes ten *MKK* genes, and *AtMKK1* and *AtMKK2* are very similar to each other^23^. To determine whether *StMKK1* is redundant in potato, we identified all MKK proteins in potato, tomato, and *N. benthamiana* and performed a phylogenetic analysis with *Arabidopsis* MKKs (Supplemental Fig. 1). Consistent with a previous study, four clades of MKKs were found in *Arabidopsis* and in these three *Solanaceae* plants. An ancient duplication event in clade A resulted in the formation of two distinct subclades of MKKs, MKK1/2, and MKK6. Additionally, several recent duplication events of *MKK* genes were found within both *N. benthamiana* and *Arabidopsis*, while no recent duplication was found in *Solanum*, i.e., potato and tomato. Therefore, it is clear that only one *MKK1* gene is present in the potato genome. Close examination of the MKK1 proteins of *Arabidopsis* and potato and tomato revealed that StMKK1 has 11 conserved subdomains and a conserved phosphorylation motif (S/T-xxxxx-S/T) in the activation loop (Supplemental Fig. 2). These data suggest that there is only one typical MKK1 gene in potato.

### StMKK1 negatively regulates plant immunity to *Phytophthora* pathogens

**Fig. 1:**
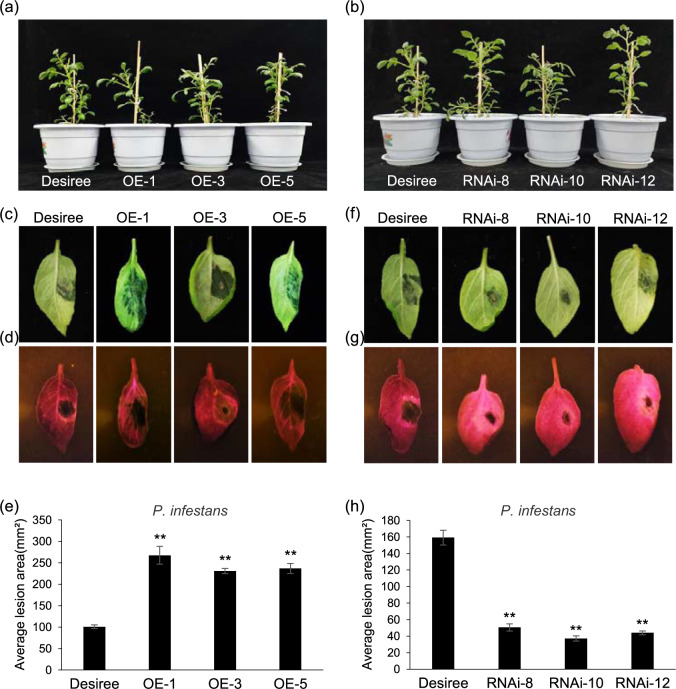
StMKK1 negatively regulates potato resistance to *P. infestans*. The morphology of *StMKK1* overexpression **(a)** and RNAi **(b)** transgenic potato plants. Representative images of Desiree- and *StMKK1*-overexpressing transgenic potato leaves with *P. infestans* lesions under natural light **(c)** and blue light **(d)**. **e** Statistical analyses show that overexpression of *StMKK1* in potato significantly increases *P. infestans* lesion areas compared to those in the wild-type Desiree. Representative leaf images of Desiree and *StMKK1* RNAi transgenic potato leaves with *P. infestans* lesions under natural light **(f)** and blue light **(g)**. **h** Statistical analyses show that silencing *StMKK1* in potato significantly decreases *P. infestans* lesion areas compared with those in Desiree. All leaves were infected with zoospores of *P*. *infestans* isolate Pi14-3-GFP and photographed at 4 dai. Each inoculation test was repeated three times with similar results. In **(e)** and **(h)**, error bars show the standard deviations from 16 replicates, and two-sided *t*-tests were used to assess significance: **, *p* < 0.01

Previously, we showed that StMKK1 negatively regulates *N. benthamiana* resistance to *P. infestans*. To investigate the role of StMKK1 in potato immunity, we analyzed the expression patterns of *StMKK1* upon *P. infestans* infection and SA treatment. The results showed that *StMKK1* expression was induced at the early infection stages during *P. infestans* infection (Supplemental Fig. 3a). Since SA plays an essential role in plant immunity, we checked the expression patterns of *StMKK1* after SA treatment, and the results showed that *StMKK1* was also induced after SA treatment (Supplemental Fig. 3b). These results suggested that StMKK1 plays a role in plant defense responses against *P. infestans* and the SA-related signaling pathway. To further confirm this, we analyzed stable potato transformants overexpressing *GFP-StMKK1* and silencing *StMKK1* (StMKK1-RNAi). The expression levels of *StMKK1* were significantly upregulated in the different overexpression (OE) transgenic plants and downregulated in the RNAi transgenic plants, as determined by qRT-PCR (Supplemental Fig. 4). The *StMKK1* OE plants, as well as the *StMKK1*-silencing plants (RNAi lines we constructed previously)^24^, showed no morphologically distinct phenotypes compared to the wild-type plant Desiree (Fig. 1a, b).

To analyze the roles of StMKK1 in potato immunity, detached leaf assays were performed for *StMKK1* OE and RNAi lines. The middle leaves of 5-week-old potato transformants were detached and inoculated with a *P. infestans* zoospore suspension. At 4 days after inoculation (dai), lesion development was photographed, and lesion diameters were measured^25^. The results showed that *StMKK1* OE lines developed larger lesions (Fig. 1c–e), while the RNAi lines developed smaller lesions than the control (Fig. 1f–h). These results indicate that StMKK1 negatively regulates plant immunity to the late-blight disease pathogen *P. infestans* in potato, similar to our previous findings in *N. benthamiana*^26^.

To investigate whether StMKK1 plays similar roles in plant immunity to other oomycete pathogens, we transiently expressed *GFP-StMKK1* and *GFP-GUS* in *N. benthamiana* leaves and inoculated the leaves with *Phytophthora parasitica* zoospores. Lesion diameters were measured at 3 dai, and the results showed that *GFP-StMKK1*-expressing leaves developed significantly larger lesions than control leaves (Supplemental Fig. 5). Taken together, these results indicate that StMKK1 accelerates the infection of *Phytophthora* pathogens and acts as a negative regulator of plant resistance against *Phytophthora* pathogens.

### StMKK1 negatively regulates potato resistance to the bacterial wilt pathogen *Ralstonia solanacearum*

**Fig. 2:**
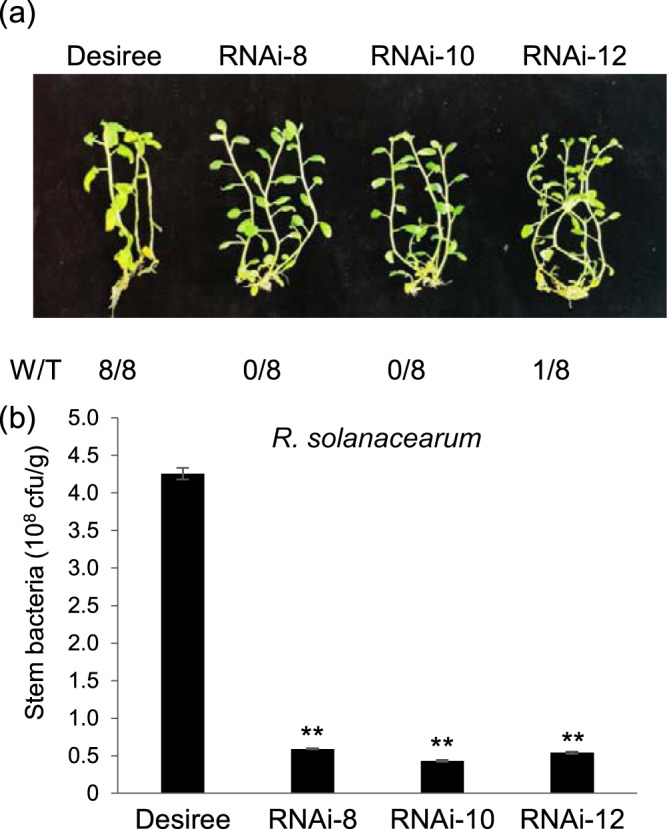
Silencing of *StMKK1* increases potato defense against *Ralstonia solanacearum*. **a** Representative images showing wilt symptoms in the wild-type Desiree and *StMKK1* RNAi transgenic lines. W/T represents the number of wilting plants with respect to the total number of infected plants. **b** Statistical analysis of the amount of bacteria in plant stems showed significantly reduced bacterial colonization in *StMKK1* RNAi lines compared with that in Desiree. Two-week-old potato plants were infected by the hydroponic infection method and photographed at 5 dai. The test was repeated three times with similar results. Error bars show the standard deviations from eight replicates. Two-sided *t*-tests were used to assess significance: **, *p* < 0.01

To test the role of StMKK1 in potato bacterial wilt disease, three independent transgenic lines, RNAi-8/10/12, were grown in liquid MS medium for 2 weeks before transformation into distilled tap water comprising 1×10^8^ cfu/mL *R. solanacearum*. The wilting symptoms were photographed, and the growth of bacteria was checked at 5 dai. As shown in Fig. 2, the control plants developed clear wilting symptoms, while *StMKK1*-silenced lines developed almost no symptoms. The quantification of bacterial growth confirmed our observation that the *StMKK1*-silenced plants contained significantly fewer bacteria than the control plants. This result indicates that StMKK1 also negatively regulates plant immunity to the bacterial wilt pathogen *R. solanacearum*. Similarly, in cotton, it was reported that GhMKK1 negatively regulates plant resistance to *R. solanacearum*^18^.

### StMKK1 enhances potato resistance to the fungal pathogen *Botrytis cinerea*

**Fig. 3:**
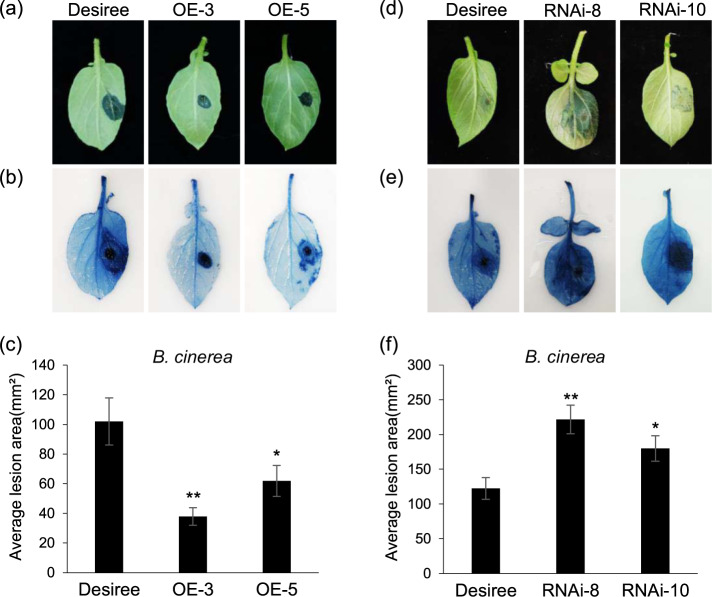
StMKK1 positively regulates potato resistance to *Botrytis cinerea*. Representative leaf images of *StMKK1* overexpression transgenic lines before **(a)** and after **(b)** trypan blue staining showing lesions of *B. cinerea* at 2 dai. **c** Bar graph showing that overexpression of *StMKK1* in potato significantly reduces *B. cinerea* lesion areas compared to that in the wild-type Desiree. Representative leaf images of *StMKK1* RNAi transgenic lines before (**d**) and after **(e)** trypan blue staining showing *B. cinerea* lesions developed at 2 dai. **f** Bar graph showing that silencing of *StMKK1* in potato significantly increases *B. cinerea* lesion areas compared to that in Desiree. All leaves were infected with *B. cinerea* B05.10. Each inoculation test was repeated three times with similar results. In **(c)** and **(f)**, error bars show the standard deviations from 16 replicates, two-sided *t*-tests were used to assess significance: *, *p* < 0.05, **, *p* < 0.01

To test the role of StMKK1 in response to the necrotrophic fungal pathogen *B. cinerea*, two independent transgenic lines of *StMKK1* OE-3/5 and RNAi-8/10 were used for infection. Middle leaves of 5-week-old plants were harvested and inoculated with *B. cinerea*. Lesion development was photographed, and the lesion diameters were measured at 2 dai. The results show that the *StMKK1* OE-3/5 lines developed smaller lesions (Fig. 3a–c), while the RNAi-8/10-silenced lines developed larger lesions than the control plants (Fig. 3d–f). These results showed that StMKK1 positively regulates potato resistance to the necrotrophic plant pathogen *B. cinerea*. This is in contrast to the hemibiotrophic and biotrophic pathogens *P. infestans* and *R. solanacearum*, for which StMKK1 negatively regulates potato immunity.

Many genes are reported to play dual roles in plant immunity; on the one hand, these genes contribute to plant susceptibility to biotrophic pathogens, and on the other hand, they promote plant resistance to necrotrophic pathogens^27,28^. There are two possibilities for this phenomenon. First, plant PTI responses result in the activation of reactive oxygen species (ROS) bursts and immune-related gene expression, which in some cases, leads to plant cell death that is unfavorable for biotrophic pathogens, as they require a biotrophic environment for disease development. However, necrotrophic plant pathogens use plant cell death responses to kill the host cell for proliferation^29^; thus, plants express an opposite response to these pathogens. Second, different plant hormones respond differently to biotrophic/hemibiotrophic or necrotrophic pathogens. For example, SA positively regulates a large portion of the plant immune response to biotrophic and hemibiotrophic plant pathogens^30,31^, while it negatively regulates plant immunity to necrotrophic pathogens^32^.

### StMKK1 inhibits potato PTI responses

**Fig. 4:**
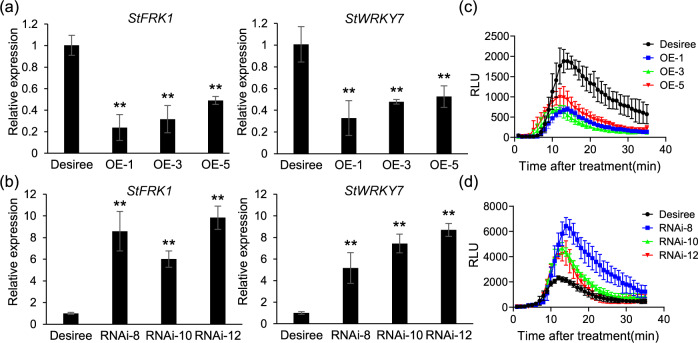
StMKK1 suppresses plant PTI responses. qRT‐PCR analysis showing the expression of the PTI marker genes *StFRK1* and *StWRKY7* in *StMKK1* overexpression **(a)** and *StMKK1* RNAi transgenic potato lines **(b)**. Total RNA was extracted from 40 µM flg22-treated leaves of the corresponding plants. *StActin* was used as a reference gene in potato. *StFRK1* and *StWRKY7* gene expression levels in Desiree were set to 1. One-sided *t*-tests were used to assess significance: **, *p* < 0.01. Flg22‐induced ROS production was measured in *StMKK1* overexpression **(c)** and *StMKK1* RNAi transgenic potato lines **(d)**. The wild-type Desiree was used as a control. Ten microliters of flg22 was used before the measurement of ROS production. RLU represents relative luminescence units. Data were analyzed by GraphPad Prism 6.0. Each experiment was repeated three times with similar results. Error bars show the standard deviations from three technical replicates

To further investigate the mechanism by which StMKK1 regulates plant immunity, we checked the PTI responses in *StMKK1* OE-1/3/5 and RNAi-8/10/12 lines. The transgenic plants were infiltrated with flg22, and PTI-related gene expression and ROS bursts were detected. As shown in Fig. 4a, c, the gene expression of *StFRK1* and *StWRKY7* was reduced and the flg22-triggered ROS burst was suppressed in *StMKK1* OE-1/3/5 plants. However, the RNAi-8/10/12 lines showed enhanced expression of PTI-related genes and induction of an ROS burst (Fig. 4b, d). These results indicate that StMKK1 negatively regulates plant PTI responses.

### StMKK1 negatively regulates SA-related immunity

**Fig. 5:**
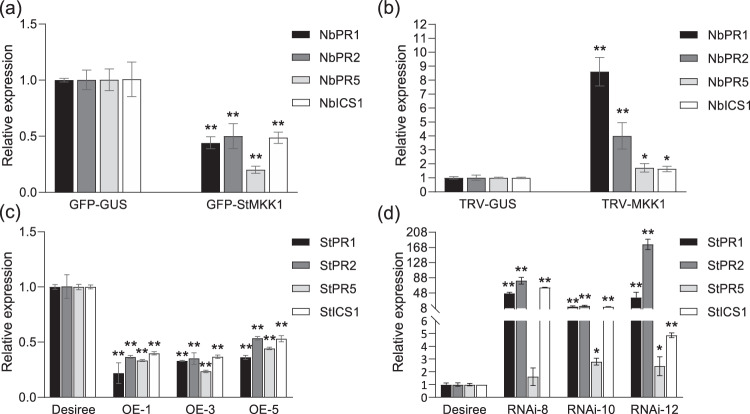
StMKK1 negatively regulates the expression of SA marker genes. The relative expression of SA marker genes in *N*. *benthamiana* expressing *GFP-GUS* and *GFP-StMKK1* **(a)**, TRV-*GUS* and TRV-*MKK1* **(b)**, and in *StMKK1* overexpression **(c)** and *StMKK1* RNAi transgenic potato lines **(d)**. Total RNA was extracted from uninfected leaves of the corresponding plants. SA marker genes *PR1*, *PR2*, *PR5,* and *ICS1* were examined. *NbActin* and *StActin* were used as reference genes in *N*. *benthamiana* and potato, respectively^45^. The expression levels of SA marker genes in GFP-GUS, TRV-GUS, and Desiree were set to 1, respectively. One-sided *t*-tests were used to assess significance: *, *p* < 0.05, **, *p* < 0.01. Error bars represent the standard deviations from three biological replicates

In *Arabidopsis*, the *Atmkk1/2* mutant shows enhanced salicylic acid (SA)-related disease resistance^33^. To reveal the role of potato StMKK1 in the SA-related defense response, we analyzed the effect of StMKK1 on SA-responsive gene expression. *StMKK1* was transiently expressed in *N. benthamiana* leaves for 2 days before harvest to assess SA-responsive gene expression. The results showed that the expression of *NbPR1*, *NbPR2*, *NbPR5,* and *NbICS1* was significantly repressed in *StMKK1*-expressing leaves compared with that in the *GFP-GUS* control (Fig. 5a). Moreover, in *NbMKK1/2*-silenced *N. benthamiana* plants, the expression of *NbPR1*, *NbPR2*, *NbPR5,* and *NbICS1* was induced significantly compared to that in control plants (Fig. 5b), which again indicates its role in the regulation of SA-related plant immunity. Consistently, qRT-PCR data in potato indicated that the expression of *StPR1*, *StPR2*, *StPR5,* and *StICS1* was repressed in *StMKK1* overexpression lines (Fig. 5c) and induced in *StMKK1* RNAi lines (Fig. 5d).

In *Arabidopsis*, the MEKK1−MKK1/2−MPK4 cascade was monitored by the NB-LRR protein SUMM2^33,34^, and mutation of MKK1/2 resulted in the activation of SUMM2 and subsequently led to the activation of SA-related immunity. Consequently, *Arabidopsis* mkk1/2 and mpk4 mutants showed lesion mimic phenotypes^33,35^. However, in both *N. benthamiana* and potato, silencing *StMKK1* did not alter plant growth; thus, we hypothesized that solanaceous plants may not have a functional *SUMM2* gene in their genome. It is likely that StMKK1 uses other mechanisms to repress SA-related immunity. Taken together, our results and previous studies have shown that StMKK1 negatively regulates the PTI response and SA-dependent immunity. It was reported that SA activates both PTI and ETI responses, which subsequently activate MAPK cascade signaling. Thus, it is not surprising to find that SA activated StMKK1 gene expression (Supplemental Fig. 3). Since plant MAPK cascades regulate complicated cellular processes, including plant immunity, the magnitude of MAPK cascade activation must be accurately regulated to avoid overactivation of plant immunity, which likely leads to the inhibition of plant development. For example, it was reported that the overexpression of *N. benthamiana* constitutively activated NbMEK2^DD^^36^, and Arabidopsis AtMKK7 and AtMKK9 induced plant cell death^37^. Thus, to repress the overactivation of MAPK cascades, plants have evolved protein phosphatases to dephosphorylate the MAPK cascade^38,39^. In our study, we found that StMKK1 negatively regulates the PTI response and SA-dependent immunity, and it is likely that plants employ StMKK1 to avoid the overactivation of immunity.

## Conclusion

In summary, we showed that overexpression of *StMKK1* in potato inhibits plant resistance to *P. infestans* and *R. solanacearum* while enhancing plant resistance to *B. cinerea*, likely by suppressing plant PTI and SA-related immunity. Silencing MKK1 enhances plant resistance to colonization by the hemibiotrophic *P. infestans* and biotrophic *R. solanacearum,* but reduces plant resistance to the necrotrophic *B. cinerea*. Our results showed that StMKK1 plays different roles in potato resistance against biotrophic and necrotrophic pathogens by negatively regulating PTI and SA-related signaling pathways.

## Materials and methods

### Agroinfiltration

GFP-StMKK1 and StMKK1-pART27-RNAi plasmids were constructed as described previously^24,26^. *Agrobacterium* strain C58C1 carrying GFP-StMKK1 or GFP-GUS plasmids was cultured in liquid LB medium with appropriate antibiotics at 28 °C. Two days later, the Agrobacterium cells were resuspended in infiltration buffer (10 mM 2-(N-morpholino) ethanesulfonic acid (MES), 10 mM MgCl_2_, and 200 mM acetosyringone, pH 5.6) to an OD600 of 0.3 and kept at room temperature for 1 h before infiltration into *N. benthamiana* leaves.

### Potato transformation

The StMKK1-RNAi lines were described previously^24^. *Agrobacterium tumefaciens*-harboring pART27-StMKK1 was transformed into potato cv Desiree by stem segment transformation as described previously^40^. The rooted transformants grown on MS medium supplemented with vitamins, 100 mg/L kanamycin, and 30 g/L sucrose were transferred to a new MS medium without kanamycin at 23 °C with a 16/8 h day/night cycle. Three weeks later, the transformants were transferred to plastic pots containing potting soil in a climate chamber at 25 °C with a 16/8 h day/night cycle. For both overexpression and RNAi constructs, more than five independent transformants were obtained and confirmed by PCR with the forward primer of the 35S promoter and the gene-specific reverse primer of *StMKK1* (Supplemental Table 1). For each transformant, more than three biological replicates were grown for further investigations.

### Pathogen strains and growth conditions

*P*. *infestans* isolate 14-3-GFP was grown on rye and sucrose agar (RSA) plates at 18 °C in the dark for approximately 2 weeks before zoospores were collected. 14-3-GFP is a *GFP*-expressing transformant of *P. infestans* H30P02 and was shown to reach a 100% infection efficiency on Desiree in a previous study^25^. *P. parasitica* was grown on 5% carrot juice agar (CA) plates at 23 °C in the dark for 4−5 days, and the zoospores were prepared as described previously^41^. *Botrytis cinerea* strain B05.10 was grown on potato dextrose agar (PDA) plates in the dark at 23 °C for 3−4 days before spores were collected. The *R. solanacearum* strain GMI1000 was grown in liquid LB medium overnight at 28 °C in a shaker.

### Plant growth condition and pathogen infection assays

Potato and *N. benthamiana* plants were grown in a climate chamber with a 16/8 h day/night cycle at 25 °C. Four- to five-week-old *N. benthamiana* plants and 5-week-old potato plants were used for infection assays. Zoospores from *P. infestans* isolate 14-3-GFP were collected as described previously^26^. Detached leaf assays were performed by inoculating 10-μL zoospore suspensions containing 1000 zoospores onto one potato leaflet. The inoculated leaves were kept in moisture in the dark at 18 °C and the lesion diameters were measured at 4 dai. For *B. cinerea* inoculation, the spore suspension was prepared as described previously^42^, and for each leaf, a 2-μL spore suspension that contained approximately 2000 spores was used. The inoculated leaves were kept at room temperature, and at 2 dai, the lesion diameters were measured. For *R. solanacearum* infection, *R. solanacearum* overnight cultures were collected and washed two times before dilution with distilled tap water to an OD600 of 0.1. Two-week-old potato plants were inoculated with *R. solanacearum* suspensions using the method described previously^43^. Wilting symptoms were observed at 5 dai, and the number of bacteria in the aerial parts of the infected plants (cfu/fresh weight) was counted as described previously^43^. For *Phytophthora parasitica* infection, the GFP-tagged strain *Pp1121* was used. *P. parasitica* was cultured, and infection assays were performed as described previously^41^, and the lesion diameters were measured at 3 dai.

### Quantitative real-time (qRT)-PCR

Total RNA was isolated from transgenic potato lines using TRIzol reagent (Invitrogen). First-stranded cDNAs were synthesized, and qRT-PCR was performed as described^26^ using the appropriate primer pairs shown in Supplemental Table 1. *StMKK1* expression levels in different transgenic lines were quantified using the 2^ΔΔ^Ct method, and the potato gene *Actin* was used for normalization. Additionally, the expression of the *N. benthamiana* gene *Actin* was used for normalization.

### Flg22 treatments, ROS production analysis, and salicylic acid treatments

The 10 µM flg22-treated leaves were subsequently used for ROS production analysis as described previously^44^. To analyze the transcript level of *StMKK1* under SA treatment, SA was dissolved in ethanol, and 10 mM SA solution was sprayed onto 2-month-old potato leaves. Leaves were harvested at 1, 3, 6, 12, and 24 h after SA spraying for RNA extraction.

### Western blotting

Samples expressing GFP-StMKK1 and GFP-GUS were extracted using lysis buffer as described previously^26^. Sodium dodecyl sulfate-polyacrylamide gel electrophoresis (SDS-PAGE) was performed to detect the proteins. Antibody (anti-GFP, goat anti-rabbit) was used according to descriptions given in the manual.

### Accession numbers

Accession numbers of StMKK1 are as follows: Sotub12g010200.1.1. NbMKK1/2: Niben101Scf02790g03012.1, Niben101Scf13387g00027.1, Niben101Scf10103g03014.1, Niben101Scf00611g07010.1.

## Supplementary information

Supplemental information revised
