# Supplementary material for: The role of the MAP kinase−kinase protein StMKK1 in potato immunity to different pathogens
Source: Hortic Res. 2021 Jun 1;8:117. doi: 10.1038/s41438-021-00556-5 (PMC8167122; doi:10.1038/s41438-021-00556-5)
Supplement: Supplementary file 1 — Supplemental information revised [file 41438_2021_556_MOESM1_ESM.docx]

**Supplemental Figure 1.** Phylogeny of the MKK proteins in Solanaceae and *Arabidopsis*. The homologs of *Arabidopsi*s MKK in potato (*Solanum tuberosum*), tomato (*Solanum lycopersicum*), and *Nicotiana benthamiana* were retrieved from the Solanaceae genomics database (https://solgenomics.net). All the MKK proteins were aligned with M-Coffee, and the phylogenetic relationship was reconstructed by maximum likelihood with PhyML. Circles on the branches, from green to red, represent the SH-like support values of the corresponding branches. A red circle indicates the branch support over 0.5. The tree was mid-rooted, and the root was indicated by a black dot.

**Supplemental Figure 2.** Amino acid sequences alignment of StMKK1 and its orthologues in *Arabidopsis* and tomato. Identical and similar amino acids are shaded in black gray, respectively. The conserved subdomains are shown with roman numbers (I–X) on the bottom. The S/T-xxxxx-S/T motif is marked with stars. The amino acid sequence alignment was generated with DNAMAN v6.0.

**Supplemental Figure 3.** The expression pattern of *StMKK1* on the treatment of *P. infestans* and salicylic acid (SA). The transcript levels of *StMKK1* during *P. infestans* infection (a) and SA treatment (b). RNAs were obtained from potato leaves sprayed with zoospores of *P. infestans* and potato seedlings that treated with 10 mM SA. qPCR was performed to examine the transcript level of *StMKK1* and *StActin* was used as a reference gene. Error bars represent the standard deviations from three biological replicates.

**Supplemental Figure 4.** The expression levels of StMKK1 in transgenic overexpression lines and RNAi lines. *StMKK1* is up-regulated significantly (a) in three independent transgenic overexpression lines (OE-1, OE-3 and OE-5) and down-regulated significantly (b) in three independent transgenic RNAi lines (RNAi-8, RNAi-10 and RNAi-12) compared with the wild type Desiree. Total RNAs were obtained from 3-week old potato leaves. *StActin* was used as a reference gene. Error bars represent the standard deviations from three technical replicates.

**Supplemental Figure 5.** Expression of *StMKK1* in *N*. *benthamiana* reduces plant resistance to *Phytophthora paracitica* compared with the control. Representative leaf images of *N*. *benthamiana* expressing *GFP-GUS* and *GFP-StMKK1* with *P.* *parasitica* lesions under natural light (a) and blue light (b). (c) Statistical analysis indicates the lesion areas in *StMKK1*-expressed leaves are significantly bigger than that in control leaves. (d) Immuno-detection of proteins from *N*. *benthamiana* expressing *GFP-StMKK1* and *GFP-GUS*.

**Supplemental Table 1.** Sequences of the primers used in this study.

| ***Primer Name*** | ***Primer Sequence (5'-3')*** |
| --- | --- |
| StMKK1EcoR1F | CCGGAATTCATGAAGAAAGGATCTTTTGCTCC |
| StMKK1Xba1R | GCTCTAGATAGCTCAGTAAGTGTTGCCAATG |
| StMKK1RNAiEcoRIF | GGAATTCTGCTCGAGTGTGCAACAGGTC |
| StMKK1RNAiKpnIR | GGGGTACCTAGCTCAGTAAGTGTTGCCAATG |
| StMKK1RNAiXbaI F | GCTCTAGATGCTCGAGTGTGCAACAGGTC |
| StMKK1RNAiHindIIIR | CCCAAGCTTTAGCTCAGTAAGTGTTGCCAATG |
| StActin qPCRF | AGCACCCTGTTCTGCTCACT |
| StActin qPCRR | GCACAGCCTGAATAGCAACA |
| StMKK1-transgeneqPCRF | TTGCTAATCAATCACAGAGGTG |
| StMKK1-transgeneqPCRR | TGTCGCTTTTGTAATCATAGGC |
| StFRK1-qPCRF | TATAGCTTCGGAGTCGTGC |
| StFRK1-qPCRR | TCATAGGTTCCATCTAGCATG |
| StWRKY7-qPCRF | CCAACTGGAAGCAACAACAA |
| StWRKY7-qPCRF | CCTGATTAGAATGATTAGCCAACA |
| NbActin-qPCRF | TCCATGCTCAATGGGATACT |
| NbActin-qPCRR | TTCAACCCCTTGTCTGTGAT |
| NbPR-1qPCRF | CCGCCTTCCCTCAACTCAAC |
| NbPR-1qPCRR | GCACAACCAAGACGTACTGAG |
| NbPR-2qPCRF | AGGTGTTTGCTATGGAATGC |
| NbPR-2qPCRR | TCTGTACCCACCATCTTGC |
| NbPR-5qPCRF | AGTGGCCGAGGTAATTGTGA |
| NbPR-5qPCRR | CATTGGTCGGGCTGAATTCC |
| NbICS1-qPCRF | CAATTCCGCCATCTCTCACT |
| NbICS1-qPCRR | TGAGCATGAAGCCACTCAAG |
| StPR-1qPCRF | CTTAACCCTCACAATGCAGCTC |
| StPR-1qPCRR | AGTGTCCACATACTTTACCCGC |
| StPR-2qPCRF | TGGTGCTGGTCTTGGAAACC |
| StPR-2qPCRR | AGAATCTGATGATGGGATCAACC |
| StPR-5qPCRF | ATGGCTACAATGTCGGTATCG |
| StPR-5qPCRR | TGTTGAATTGTGCACACGCG |
| StICS1 qPCRF | AGCGTCTTGGGACAATGTGG |
| StICS1 qPCRR | TGCTCTGGAGTGTTTCCAATG |
| pKanF | CAATCCCACTATCCTTCGCA |
| pKanR | CGGTAAGGATCTGAGCTACA |
